# Rapid Gut Adaptation to Preterm Birth Involves Feeding-Related DNA Methylation Reprogramming of Intestinal Genes in Pigs

**DOI:** 10.3389/fimmu.2020.00565

**Published:** 2020-04-15

**Authors:** Xiaoyu Pan, Thomas Thymann, Fei Gao, Per T. Sangild

**Affiliations:** ^1^Comparative Pediatrics and Nutrition, Department of Veterinary and Animal Sciences, Faculty of Health and Medical Sciences, University of Copenhagen, Copenhagen, Denmark; ^2^Genome Analysis Laboratory of the Ministry of Agriculture, Agricultural Genomics Institute at Shenzhen, Chinese Academy of Agricultural Sciences, Shenzhen, China; ^3^Department of Neonatology, Rigshospitalet, Copenhagen, Denmark; ^4^Department of Pediatrics, Odense University Hospital, Odense, Denmark

**Keywords:** preterm birth, small intestine, DNA methylation, nutrition, immunity, metabolism

## Abstract

Following preterm birth, the immature gut function and immunology must rapidly adapt to cope with bacterial colonization and enteral milk feeding. We hypothesized that intestinal epigenetic changes are involved in the gut response to preterm birth and the first feeding. Using piglets as models for infants, preterm, and term pigs were fed total parenteral nutrition (TPN) or partial enteral feeding for 5 days, followed by exclusive enteral feeding with bovine milk until day 26 (weaning age). Intestinal structure, function, microbiome, DNA methylome, and gene expressions were compared between preterm and term pigs on days 0, 5, and 26 (*n* = 8 in each group). At birth, the intestine of preterm pigs showed villus atrophy and global hypermethylation, affecting genes related to the Wnt signaling pathway. Hypermethylation-associated lowered expression of lipopolysaccharide-binding protein and genes related to the Toll-like receptor 4 pathway were evident during the first 5 days of life, but most early methylation differences disappeared by day 26. Regardless, sucrase and maltase activities (adult-type brush border enzymes) remained reduced, and the gut microbiota altered (fewer *Akkermansia*, more Lachnoclostridia and Lactobacilli) until day 26 in preterm pigs. During the 0- to 5-day period, many new preterm–term methylation differences appeared, but mainly when no enteral feed was provided (TPN feeding). These methylation differences affected intestinal genes related to cell metabolism, including increased GCK (glucokinase) expression via promoter hypomethylation. In conclusion, the immature intestine has a remarkable capacity to adapt its gene methylation and expression after preterm birth, and only few preterm-related defects persisted until weaning. Early enteral feeding may be important to stimulate the methylation reprogramming of intestinal genes, allowing rapid intestinal adaptation to preterm birth.

## Introduction

Preterm birth accounts for ~10% of all live births and remains a major global health problem (1). For survival after preterm birth, the structure and function of the immature gut must rapidly adapt to the new nutritional and microbial environment. This adaptation may fail, or take some time to develop, as indicated by a high sensitivity to feeding intolerance, gut inflammation, and necrotizing enterocolitis (NEC) in preterm infants during the first weeks after birth (2, 3). Later, children born preterm may show elevated risks of neurologic and metabolic disorders (4–7), whereas persistent gut complications are less often reported. Both short- and long-term gut complications may result from a combination of three critical factors, shortened gestational age at birth, premature transition to enteral feeding, and inappropriate bacterial colonization. Thus, the optimal dietary strategy (e.g., timing, volume, and type of diet) and most appropriate bacterial colonization of the immature gut remain unknown. A better understanding of how the immature gut interacts with environmental factors, such as nutrition and microbes, is required to define how early feeding strategies can best secure optimal adaptation of the preterm infant to postnatal life.

Adequate nutrient supply is critical for growth and organ development in preterm infants, but mother's milk is often insufficient or delayed in supply during the first weeks after preterm birth. Partial or total parenteral nutrition (TPN) is often used to support nutritional intake during the first 1–2 weeks, and this helps to combat the high sensitivity to feeding intolerance and NEC (8). On the other hand, lack of milk-derived immunomodulatory and trophic factors during TPN may compromise intestinal maturation (9, 10) and lead to more systemic infections and metabolic disorders (11). Preterm pigs, an animal model of preterm birth with a full range of prematurity signs (e.g., high sensitivity to NEC, sepsis, respiratory distress, neurological impairment, metabolic disorders) (12), respond to fast increases in enteral feeding (e.g., >120 mL/kg per day within few days) with marked intestine-trophic responses, but also with a high NEC sensitivity, especially when the diet is infant formula vs. more protective milk diets, such as sow's or cow's colostrum (12–14). The NEC risk is much less when enteral feeding is advanced slowly, in parallel with parenteral nutrition (15), similar to the situation for most preterm infants. There are differences in transfer of passive immunity between pigs and infants (e.g., immunoglobulin transferred postnatally via the gut in piglets vs. transplacental transport before birth in infants), but these differences do not appear to explain differences in NEC sensitivity (16). Even small volumes of formula may be detrimental in both preterm infants and pigs, and gradual feeding with natural milk or colostrum products may benefit intestinal maturation (15, 17, 18). Early enteral feeding (ENT) with small volumes is therefore recommended for preterm infants (10) despite that its diet dependency and the benefits vs. risks remain debated. It is therefore important to investigate the intestinal molecular functions affected by early TPN and early supplemental ENT feeding to understand the biological pathways and mechanisms of gut adaptation in preterm neonates. In an ongoing clinical trial in very preterm infants (ClinicalTrials.gov identifier NCT03085277), ENT with small volumes of bovine colostrum is being investigated.

As one of the important epigenetic mechanisms of tissue adaptation, DNA methylation plays a key role in sensing environmental exposures, thereby regulating transcription and cellular function during development (19). Because of the shortened gestation, intestines of preterm neonates may be subject to an accelerated adaptation after birth to adjust cellular functions, together with distinct DNA methylation profiles that reflect developmental immaturity at birth and postnatal exposure to enteral milk and microbes. We previously showed that formula feeding and bacterial colonization have marked short-term effects on intestinal DNA methylation in preterm pigs (18, 20), but it is not known if such changes are stable more long-term and ENT-dependent. Preterm–term comparisons of the intestinal methylome with advancing postnatal age help to elucidate if the phenotypic and molecular differences observed at birth may have long-term consequences for gut development in preterm neonates.

We hypothesized that the intestine of preterm neonates has a distinct epigenetic signature at birth and that DNA methylation changes in the postnatal period depending on the presence of ENT stimulation, to help adapt the immature intestine to postnatal life. Cesarean-delivered preterm or term pigs were euthanized for tissue collection shortly after birth or fed TPN or gradually increasing amounts of enteral nutrition (ENT) using bovine colostrum for 5 days, followed by transition to the same milk diet until day 26 (weaning age). The two feeding regimen, both of which minimize NEC risk (15), allowed us to examine how the immature intestine responded to the presence of enteral nutrition without confounded by NEC. Intestinal phenotypes (including morphology, digestive and absorptive function, microbiota composition), genome-wide DNA methylation, and targeted gene expression were compared between preterm and matched term pigs.

## Materials and Methods

### Animal Experimental Procedure

All animal procedures were approved by the Danish National Committee on Animal Experimentation. One hundred sixty-eight piglets from eight sows (Danish Landrace × Large White × Duroc) were delivered by cesarean section at full term (day 118 or 100% gestation, n = 56 pigs from three sows) or preterm (day 106, 90% of gestation, *n* = 112 pigs from five sows) (21, 22). Based on immaturities in the intestine and other organs (e.g., lungs, liver, kidney, immunity), preterm 90% gestation pigs can be considered a relevant model for corresponding aspects in human infants delivered at ~70% gestation (12). Immediately after cesarean section, all pigs were transferred to our piglet neonatal intensive care unit and reared in individual incubators. Within 3 h of delivery, all pigs were fitted with orogastric and umbilical arterial catheters. Preterm and term pigs were randomly assigned to be euthanized immediately after birth (*n* = 8, sodium pentobarbital, 200 mg/kg, intraarterial) or to receive total parental nutrition (TPN) or parenteral nutrition plus supplemental enteral nutrition (ENT) for 5 days. Total parenteral nutrition–treated pigs were given parenteral nutrition with gradually increasing volume (from 96 mL/kg per day on day 1– 114 mL/kg per day on day 5). Enteral feeding–treated pigs were given bovine colostrum with gradually increasing volume (from 16 mL/kg per day on day 1–64 mL/kg per day on day 5), accompanied by a reduction in parental nutrition, such that the two dietary regimens both provided similar fluid volumes and were isoenergetic. In this setting, parental nutrition was infused continuously, while bovine colostrum was given every 3 h using the orogastric catheter. Importantly, the volume progression (16–64 mL/kg per day) was relatively slow, representing a careful approach to advance enteral nutrition.

On day 5, TPN and ENT pigs were randomly assigned to be euthanized for tissue collection or transitioned to full enteral nutrition with cow's milk, a gut-protective diet for preterm pigs (23), until euthanasia on day 26. For the latter ones, parental nutrition was discontinued on day 5, and pigs started to receive full enteral nutrition via a trough until the end of the study (8–10 feedings per day). During the period when pigs learned drinking milk from the trough (2–3 days), any remaining milk left in the trough was given to the pigs via the orogastric catheter. Therefore, during transition from TPN or partial ENT to full ENT, all pigs received the preplanned milk amount. All animals were reared individually throughout the study. Incubators with controlled ventilation and heating (from 0 to 5 days), larger home boxes (from 5 to 12 days), and even larger home cages (from 12 to 26 days) were used. All details of animal procedures were described previously (21, 22).

**Figure 1:**
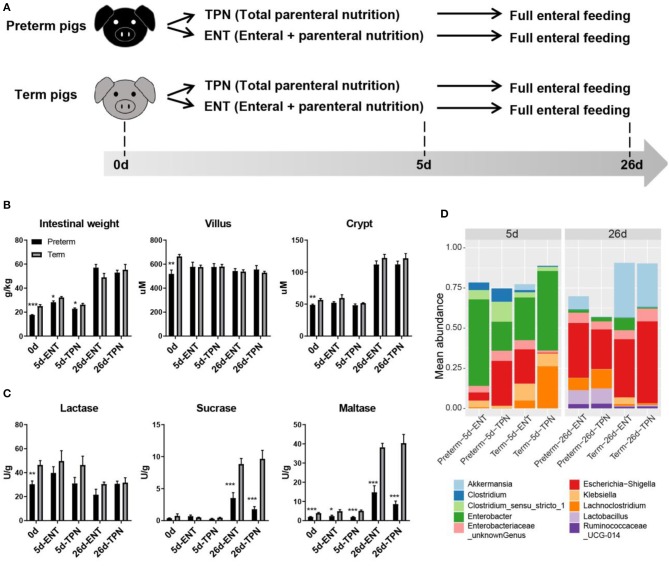
Study design and intestinal phenotypes of preterm and term pigs. **(A)** Study design showing feeding regimens and sampling time points of preterm and term pigs (*n* = 8 from two to three litters in each group). **(B)** Intestinal morphology (i.e., weight, villus height, crypt depth), **(C)** digestive function (i.e., brush border enzymes activities), and **(D)** colon bacterial abundance between preterm and term pigs were compared. The top 10 abundant genera were plotted. Differential colon bacterial abundance was detected in *Akkermansia, Lachnoclostridia*, and *Lactobacilli* on day 26. Values in barplots **(B,C)** are presented as mean ± SEM. ^*^P < 0.05, ^**^P < 0.01, ^***^P < 0.001.

Consequently, intestinal tissues were collected on days 0, 5, and 26, from a total of 10 groups of pigs. Eight pigs from each group were randomly selected for this study (Figure 1A, total *n* = 80). Shortly before euthanasia, intestinal permeability was measured by the lactulose-mannitol technique, glucose absorptive capacity by performing a galactose absorption test, plasma levels of glucose-dependent insulinotropic polypeptide (GIP) by radioimmunoassay, and diarrhea was evaluated by a diarrhea score, as described previously (22). Immediately after euthanasia, two 1-cm pieces of the middle of small intestine (jejunum, 50% along the length) were collected, snap frozen in liquid nitrogen, and kept at −80°C for subsequent analysis of enzyme activities, DNA methylome, and gene expression. Considering our aim to characterize the overall intestinal methylation differences, involving all interacting cell populations, we decided to analyze full-thickness intestine. Two additional full-thickness 1-cm sections of the middle intestine were fixed in 4% paraformaldehyde for later histological analysis. Luminal content from colon was collected for later microbiota analysis.

### Intestinal Morphology and Enzyme Activity Analyses

To evaluate the mucosal morphology, two paraformaldehyde-fixed jejunum samples were embedded in paraffin, sectioned, mounted on slides, and stained with hematoxylin and eosin before measuring villus height and crypt depth as described previously (23). For each pig, villus height and crypt depth were measured from 10 representative well-oriented villus-crypt axes by ImageJ software, National Institutes of Health, USA. The mean of 10 villus heights and crypt depths were used as representative value for one pig. To estimate the proportion of epithelial cells in the middle small intestine, immunohistochemistry (IHC) using the epithelial cell marker (cytokeratin) was performed, and the proportion of the positive staining for cytokeratin in cross-sectional areas was calculated by the IHC toolbox in ImageJ. Finally, activities of brush border enzymes (lactase, maltase, and sucrase) were analyzed in homogenates of the middle intestinal tissues by spectrophotometry and were expressed as units per gram of wet tissue, as described previously (20, 22).

### Reduced Representation Bisulfite Sequencing

Genomic DNA from the middle intestinal tissues was extracted using DNeasy Blood & Tissue Kit (Qiagen, Hilden, Germany) and was subjected to reduced representation bisulfite sequencing (RRBS), as previously described (24). Raw sequencing data were processed by the Illumina base-calling pipeline. Low-quality reads that contained more than 30% N's or >10% of the sequence with low-quality value (quality value <20) per read were omitted from the data analysis. Bisulfite sequence mapping program was used for sequence alignment to the Ensembl pig reference genome (Sscrofa10.2). Methylation level of individual cytosine was calculated as the ratio of sequenced depth of methylated cytosine to the total sequenced depth of the individual cytosine. One of 80 samples failed in RRBS and was excluded in DNA methylation analysis.

### Gene Expression Analysis

Gene expression was analyzed as previously described (25). Briefly, total RNA was isolated from middle intestinal tissues (*n* = 8 in each group), using the RNeasy Mini Kit (Qiagen), and cDNA was synthesized using 2 μg total RNA by High-Capacity cDNA Reverse Transcription Kit (ThermoFisher, Waltham, MA, USA) according to the manufacturer's instructions. Primers for real-time quantitative polymerase chain reaction (RT-qPCR) were designed using Primer-BLAST. Real-time qPCR analysis was performed using QuantiTect SYBR Green PCR Kit (Qiagen) on LightCycler 480 (Roche, Basel, Switzerland), and results were analyzed according to double delta Ct method. Relative quantification of target genes was normalized to the housekeeping gene *HPRT1* and was presented as mean values ± SEM.

### Microbiota Analysis

Total DNA was extracted, and the v3–v4 hypervariable regions of the 16S rRNA sequence were amplified through PCR. The resultant amplicons were sequenced using the Illumina MiSeq system (Illumina, San Diego, CA, USA), producing paired-end reads. The raw data set containing pair-ended reads was merged, trimmed, filtered from chimeric reads, and subjected to operational taxonomic units (OTUs) clustering using the UPARSE pipeline. Representative sequences were aligned to the SILVA reference alignment (version: SILVA123). The OTU annotation results were employed to determine the microbiota composition in the colon region of each pig.

### Statistical Analysis

Comparisons of phenotypic variables (villus height, crypt depth, brush border enzyme activities, gene expressions) were made using Student *t*-test, and a two-tailed *p* < 0.05 was considered as statistically significant. Comparison of epithelial proportion was made using Mann–Whitney *U*-test, and a two-tailed *p* < 0.05 was considered as statistically significant. Differentially methylated regions (DMRs) were identified as previously described (20). Briefly, the methylation levels between the two groups were tested using the Mann–Whitney *U*-test, and false discovery rate (FDR) of DMRs was controlled at level 0.05 with the Benjamini–Hochberg procedure. For microbiome analysis, Shannon diversity differences were tested using Mann–Whitney *U*-test, and a two-tailed *p* < 0.05 was considered statistically significant. Comparisons of relative abundance were made using Mann–Whitney *U*-test, and all *p*-values were adjusted for multiple comparisons with FDR correction.

## Results

### Intestinal Morphology, Digestive Function, and Gut Microbiota Differ Between Preterm and Term Pigs

Preterm pigs had lower relative intestinal weight than term pigs at birth (17.7 ± 0.3 vs. 25.0 ± 1.3 g/kg, *p* < 0.05) and day 5 (28.3 ± 1.1 vs. 32.2 ± 0.8 g/kg for ENT, 22.8 ± 0.8 vs. 26.2 ± 1.1 g/kg for TPN, both *p* < 0.05), but not at weaning (57.1 ± 2.7 vs. 49.0 ± 3.4 g/kg for ENT, 53.0 ± 2.0 vs. 55.3 ± 4.5 g/kg for TPN, both *p* > 0.05; Figure 1B). Villus height in the middle intestine was similar among 0-, 5-, and 26-day-old pigs (586.2 ± 27.8 μm for 0 days, 577.5 ± 13.2 μm for 5 days, 541.4 ± 10.2 μm for 26 days, *p* > 0.05). Crypt depth was similar between 0- and 5-day-old pigs (52.4 ± 1.6 vs. 52.7 ± 1.5 μm, *p* > 0.05), but there was a sharp increase from days 5 to 26 (52.7 ± 1.5 vs. 117.0 ± 3.0 μm, *p* < 0.0001; Figure 1B). Only at birth did preterm pigs show shorter villi (519.0 ± 32.3 vs. 664.6 ± 16.3 μm, *p* < 0.01) and crypts (48.9 ± 1.1 vs. 56.5 ± 2.1 μm, *p* < 0.01) than term pigs. The proportion of epithelial cells (59% on average), as estimated by IHC staining of cross sections of the intestine, was similar between the preterm and term pigs (*p* > 0.05), and regardless of age. For activity of brush border enzymes in the middle intestine, lactase was high relative to sucrase and maltase at birth (37.9 ± 3.0 vs. 0.6 ± 0.2 and 2.9 ± 0.3 U/g, both *p* < 0.0001). Preterm pigs had lower lactase activity than term pigs at birth (30.4 ± 2.7 vs. 46.5 ± 3.5 U/g, *p* < 0.01; Figure 1C). Sucrase and maltase activities increased markedly from days 5 to 26 (0.5 ± 0.1 vs. 6.0 ± 0.8 U/g and 3.6 ± 0.4 vs. 25.5 ± 2.9 U/g, respectively, both *p* < 0.0001), but remained to be lower in preterm than term pigs (2.7 ± 0.5 vs. 9.3 ± 0.8 U/g and 11.7 ± 2.0 vs. 39.3 ± 2.4 U/g, respectively, both *p* < 0.001; Figure 1C). Despite their immature state at birth, preterm pigs had similar gut permeability, glucose absorptive capacity, and diarrhea scores as term pigs during the postnatal period (data not shown). No preterm or term pigs showed any signs of NEC. Analyses of the colon microbiota by 16s rRNA gene sequencing showed no difference in alpha diversity or relative abundances of genera between preterm and term pigs on day 5, due to large individual variation. However, on day 26, the preterm pigs had lower abundance of *Akkermansia* (4.0 vs. 30.7%) and higher abundance of *Lachnoclostridium* (9.6 vs. 1.1%) and *Lactobacillus* (9.1 vs. 0.7%, all adjusted *p* < 0.05), compared with term pigs (Figure 1D). Except that early ENT feeding significantly increased the relative intestinal weight in both groups on day 5 (28.30 ± 1.09 vs. 22.77 ± 0.76 for preterms and 32.23 ± 0.80 vs. 26.23 ± 1.09 for terms, both *p* < 0.001), none of the other measured phenotypic characteristics of the gut were consistently affected by ENT vs. TPN feeding.

### The Intestinal DNA Methylome Is Hypermethylated at Birth in Preterm Pigs

A total of 39.7 mio reads per sample were generated from RRBS (mean across samples). Among the reads passing QC (~33.8 mio/sample), 67.7% could be mapped to the Ensembl pig reference genome (Sscrofa10.2, Table S1). DNA methylation mainly occurred on CpG cytosines (mean methylation level, *m* = 60.4%), rather than CHG (*m* = 0.7%) or CHH cytosines (*m* = 0.6%). Thus, only CpG cytosines were analyzed thereafter. To avoid the potentially confounding influence of X chromosome inactivation on DNA methylation patterns between male and female pigs, only autosomal data were used. A total of 1,772,546 CpG cytosines detected in 10 groups of pigs were used for analysis.

**Figure 2:**
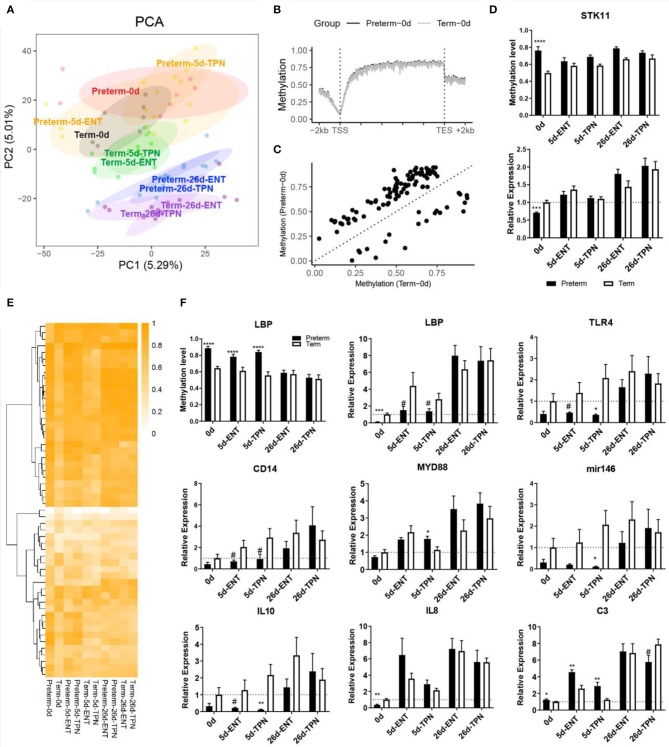
DNA methylation and gene expression difference at birth and their postnatal changes. **(A)** Scores of first two principle components from PCA are plotted, with ellipses for the 80% confidence interval for each group. Pigs with the same age after either preterm or term delivery are labeled with the same colors. **(B)** Birth DNA methylation levels along the gene bodies and 2-kb upstream of the TSS and 2-kb downstream of the TES of all Ensembl genes. **(C)** Scatterplot showing mean methylation level of DMRs in preterm and term pigs at birth. **(D)** Barplot showing promoter methylation level and mRNA expression of STK11. **(E)** Heatmap depicting methylation level of birth DMRs in postnatal period. Hierarchical clustering was performed using complete linkage method with euclidean distance. **(F)** Barplot showing promoter methylation level and mRNA expression of LBP, and mRNA expression of immunity-related genes. Values in barplots are presented as mean ± SEM. ^#^*P* < 0.1, ^*^P < 0.05, ^**^P < 0.01, ^***^P < 0.001, ^****^P < 0.0001.

Based on the individual cytosines, principal component analysis (PCA) showed that intestinal DNA methylomes were distinct for each age group. Moreover, the preterm and term groups remained separated postnatally (Figure 2A). Next, we started with a comprehensive genome-wide characterization of the intestinal CpG methylation in preterm and term pigs at birth (before any feeding). Similar to other mammalian DNA methylation landscape (26), the overall DNA methylation level of the gene body, that is, the genomic region from transcription start site (TSS) of a gene to its transcription end site (TES), was higher than that of adjacent intergenic regions (72.5 vs. 41.8%, *p* < 0.0001), and there was a marked hypomethylation (7.4%) around the TSS (Figure 2B, Table S2). The preterm intestines showed global hypermethylation compared to the term group (64.3 vs. 63.6%, paired Mann–Whitney *U*-test *p* < 0.0001; Figure 2B). A total of 103 genomic regions were identified as significantly DMRs between preterm and term pigs (Figure 2C, Table S3). There were more hypermethylated DMRs (*n* = 82) than hypomethylated DMRs (*n* = 21). These DMRs included 941 CpG cytosines, equivalent to 0.05% of all the cytosines analyzed. In preterm pigs, 76% of these cytosines showed relative hypermethylation, and 37% was highly methylated (methylation level >75%).

Among the total 103 DMRs, 59 overlapped with genic regions, particularly in introns. Fifty-five genes contained at least one DMR in either their putative promoters or the gene bodies. Genes that contained DMR in their putative promoter included BHLHA9, SLC5A10, COX17, lipopolysaccharide-binding protein (LBP), STK11, CPTP, RD3L, FGFR3, C4orf46, NAP1L5, and ZPBP. Gene ontology enrichment analysis on all the 55 genes resulted in multiple biological processes, including steroid hormone receptor activity and canonical Wnt signaling pathway (Table S4). STK11 (also called LKB1) was involved in canonical Wnt signaling pathway that is important for epithelium development (27) and possessed a DMR in the putative promoter where methylation level was higher in preterm vs. term newborns (74 vs. 50%). Consistent with that promoter methylation may suppress gene expression, the mRNA expression of STK11 was down-regulated in the preterm newborns (0.7-fold, *p* < 0.001; Figure 2D). Similar to that the villus height and crypt depth differed between preterm and term pigs only at birth, differential methylation and gene expression of STK11 were detected at birth, but not on day 5 or 26.

### Persistent Abnormal Methylation and Expression of Intestinal Genes Related to Innate Immunity

To investigate whether the observed differences of intestinal methylation between preterm and term newborns persist into the postnatal period, DMRs in 5- and 26-day-old pigs, fed either TPN or ENT during the first 5 days, were identified and compared with the 103 DMRs detected at birth. Only 6 and 28 of the 103 newborn DMRs persisted until day 5 in ENT- or TPN-feeding groups, respectively. On day 26, just before weaning, all the DMRs detected at birth were no longer different between preterm and term pigs (Figure 2E). The 34 DMRs that lasted until day 5 in preterm pigs were associated with 13 genes, of which four genes (LBP, LHPP, NR3C1, WHSC1) were identified both in the ENT and TPN groups. The remaining nine intestinal genes with preterm–term gene methylation differences that lasted until day 5 were found only among TPN-fed pigs.

Among the above four common genes containing persistent DMRs until day 5, only LBP (lipopolysaccharide binding protein) contained a DMR within its putative promoter region, showing hypermethylation in preterm pigs (89 vs. 64% at birth and 84 vs. 57% by day 5; Figure 2F). The mRNA expression of LBP was negatively correlated with its promoter methylation level (Spearman ρ = −0.54, *p* < 0.0001) and was less expressed in preterm vs. term pigs at birth (0.1-fold, *p* < 0.001) and day 5 (0.3-fold for ENT, 0.5-fold for TPN, both *p* < 0.1; Figure 2F). Lipopolysaccharide-binding protein is important for initiating Toll-like receptor 4 (TLR4) signaling, which is essential for neonatal intestinal immune tolerance (28) and was proposed to impact the risk of NEC in preterm infants (29). Thus, expression of genes involved in TLR4 signaling was also examined. Similar to LBP, both TLR4 and the accessory protein CD14 tended to be less expressed in 5-day-old preterm vs. term pigs (both 0.3-fold, *p* < 0.1). MYD88, which mediates signal transduction for TLR4, was comparable between preterm and term pigs or even up-regulated in TPN-fed 5-day-old preterm pigs (1.6-fold, *p* < 0.05; Figure 2F). Moreover, microRNA-146a and interleukin 10 (IL-10), representative for intestinal anti-inflammation, were less expressed in 5-day-old preterm pig, especially those fed TPN (0.05- and 0.06-fold, respectively, both *p* < 0.05), whereas proinflammatory cytokine IL-8 was less expressed in preterm pigs at birth (0.4 fold, *p* < 0.01) and increased to a similar level to that in term pigs on day 5 (Figure 2F). C3 that is involved in complement cascade, another component of the innate immune response, showed persistent overexpression in preterm vs. term pigs from birth (1.2-fold) to day 5 (1.8-fold for ENT, 2.3-fold for TPN, all *p* < 0.05; Figure 2F).

Another DMR was located within a CpG island in the gene body region of NR3C1 encoding for the glucocorticoid receptor (GR), which may interfere with TLR4 signaling. This DMR showed hypermethylation in preterm vs. term pigs (89 vs. 69% at birth and 90 vs. 70% on day 5), but the DNA methylation level was not correlated with gene expression (Spearman ρ = 0.15, *p* > 0.05). Except that C3 was up-regulated by ENT vs. TPN during the first week regardless of gestation (1.6-fold for preterm, 2.1-fold for term, both *p* < 0.05), no differences in DNA methylation or mRNA expression in the above genes were detected between the ENT- and TPN-treated pigs.

### Reprogramming of the Intestinal DNA Methylome in Preterm Neonates Is Feeding-Dependent

**Figure 3:**
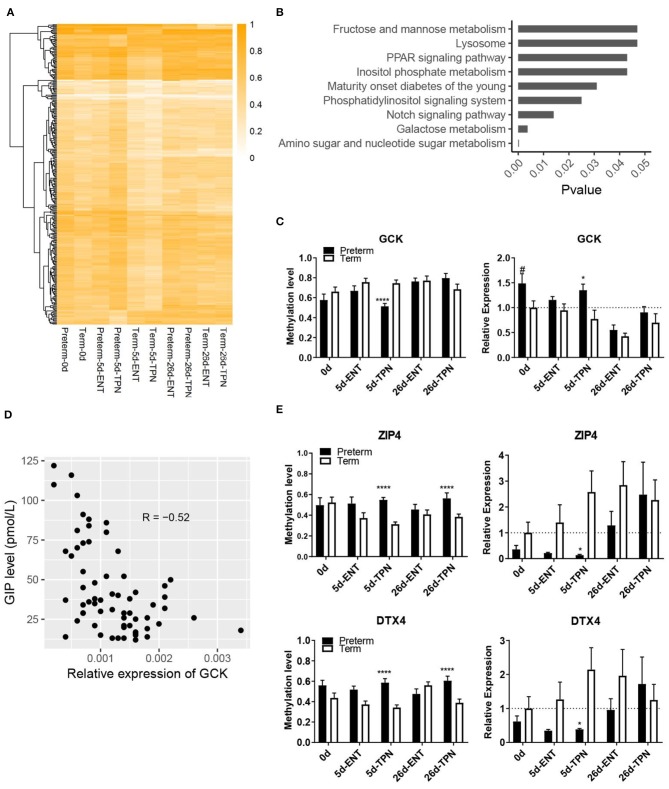
Methylation differences between preterm and term intestines after birth. **(A)** Heatmap depicting methylation level of DMRs that present in postnatal period. Hierarchical clustering was performed using complete linkage method with euclidean distance. **(B)** KEGG pathways enriched from genes with TPN-specific DMRs. **(C)** Barplots showing methylation level of the DMR and mRNA expression of GCK. Values in barplots are presented as mean ± SEM. ^#^*P* < 0.1, ^*^P < 0.05, ^**^P < 0.01, ^***^P < 0.001. **(D)** Scatterplot illustrating the correlation between mRNA expression of GCK in the small intestine and plasma GIP level. **(E)** Barplots showing methylation level of the DMR and mRNA expression of ZIP4 and DTX4. Values in barplots are presented as mean ± SEM. ^*^P < 0.05, ^**^P < 0.01, ^***^P < 0.001, ^****^P < 0.0001.

Although most of the DNA methylation differences observed at birth disappeared by day 5 and especially day 26, PCA showed persistent separation between the preterm and term groups in postnatal period (Figure 2A). A detailed look into the four postnatal groups of DMRs, that is, 5d-ENT, 5d-TPN, 26d-ENT, and 26d-TPN pigs, revealed a number of new postnatal DMRs that did not overlap with the DMRs detected at birth. On day 5, there were more new DMRs in the TPN group (*n* = 800) than in the ENT group (*n* = 60; Figure 3A, Tables S5, S6). The 800 new DMRs in the TPN group consisted of 6,767 CpG cytosines, equivalent to 0.38% of all the cytosines analyzed. Approximately 89% of the involved cytosines showed hypermethylation in the preterm group. These 800 TPN-specific DMRs overlapped with putative promoters of 54 genes and gene bodies of 255 genes.

Pathway enrichment analysis based on all the genes containing the TPN-specific DMRs revealed nine KEGG pathways being significantly enriched (Figure 3B, Table S7), including multiple metabolic pathways (e.g., galactose metabolism, amino sugar, and nucleotide sugar metabolism). Among the 20 genes involved in the above nine KEGG pathways, one gene (GCK, encoding glucokinase) was involved in multiple pathways and had a DMR located within its putative promoter region, where methylation level was correlated with gene expression (Spearman ρ = −0.54, *p* < 0.01). Glucokinase showed promoter hypomethylation (51 vs. 75%) and increased gene expression in 5-day-old TPN-fed preterm vs. term pigs (1.7-fold, *p* < 0.05; Figure 3C). This GCK expression also correlated negatively with the plasma level of GIP, a sensor for luminal glucose (30) (*p* < 0.0001, *r* = −0.52; Figure 3D). To explore additional effects of the gene methylation–regulated intestinal GCK, a protein–protein interaction analysis with the remaining 19 genes involved in the enriched pathways was performed. Result showed that GCK was directly or indirectly related to seven genes (GMPPA, GMDS, GALE, GALK1, PPARA, PPARD, and FABP1).

Next, to examine whether the new postnatal preterm–term methylation difference persisted until weaning, we searched for overlapped DMRs in 5- and 26-day groups. The result revealed only two genes (ZIP4 and DTX4), which showed no methylation difference at birth. However, hypermethylation of these two genes occurred after 5 days of TPN (but not ENT) feeding (55 vs. 31% for ZIP4, 59 vs. 34% for DTX4), and hypermethylation persisted until 26 days (56 vs. 39% for ZIP4, 61 vs. 39% for DTX4), although all pigs received full ENT from 5 to 26 days (Figure 3E). Subsequent analysis of mRNA expression of these two genes showed hypermethylation-associated down-regulation in TPN-fed preterm pigs on day 5 (0.1-fold for ZIP4, 0.2-fold for DTX4, both *p* < 0.05), but not day 26 (Figure 3E).

On day 26, the number of new DMRs in the TPN group (*n* = 31) was similar to that in the ENT group (*n* = 39), and these were associated with a similar number of genes (14 and 15 for TPN and ENT, respectively, Tables S8, S9). In both ENT and TPN groups, DMRs associated with CAMK2G, BRD3, and BAHCC1 were detected. Methylation level of DMRs in these three genes was higher (79 vs. 53% for CAMK2G, 81 vs. 53% for BRD3, and 69 vs. 40% for BAHCC1) in preterm vs. term pigs on day 26. As sucrase activity differed between preterm and term pigs only on day 26, correlation between sucrase activity and the methylation of these three genes was analyzed using all pigs. Significant negative correlation was found (all Spearman ρ < −0.6, *p* < 0.0001). In summary, the above results described a feeding-dependent DNA methylation reprogramming pattern in the neonatal intestine following preterm birth.

## Discussion

Perinatal intestinal maturation is determined by intrinsic genetic mechanisms (a predetermined “biological clock”), as well as external stimuli, including the birth process, nutrition, and microbes. When born preterm, the intestine needs to rapidly adapt to tolerate the transition from parenteral to enteral nutrition while avoiding NEC. Total parenteral nutrition or parenteral nutrition together with small volume of natural milk or colostrum is NEC-protective in preterm infants (31) and preterm pigs (15). It is critical to understand the molecular mechanisms that make the preterm intestine adapt, or fail to adapt, and characterize the subclinical differences between these two feeding regimen. Considering that DNA methylation can be affected by nutrition and plays a key role in regulating gene expression, our study provides new knowledge on feeding-dependent intestinal adaptation in preterm neonates. We show that reduced gestational age at birth (independent of other birth-related factors) is associated with a marked hypermethylation of intestinal genes that largely disappears by weaning. The two NEC-protective feeding regimens used in this study for the first 5 days (TPN, ENT) modulated the immune response in similar ways, but differed in their effects on specific genes related to intestinal metabolism. Preterm birth and the early diet differences may not have major long-lasting effects on intestinal functions, as indicated by the limited number of persistent gene methylation changes. However, it cannot be excluded that a more NEC-provocative early feeding regimen, using fast advancement of infant formula, would have more lasting effects on gene expressions and its regulation by gene methylation differences.

The global intestinal hypermethylation at preterm birth included a key gene, STK11/LKB1, which is a tumor suppressor gene in the Wnt/β-catenin pathway to regulate intestinal epithelial cells apoptosis and control cell proliferation (32). Here we showed that at preterm birth the immature intestine had shorter villus height and crypt depth, together with less expression of STK11 via promoter hypermethylation. The down-regulation of STK11 in the immature intestine may facilitate intestinal epithelial cell growth after preterm birth, and consistently, difference in the villus height and crypt depth disappeared within the first week of life. Thus, our results suggest that DNA methylation plays an important role in helping the immature intestine to adapt to postnatal life, both when fed enterally and parenterally during the first day after birth.

Intestinal hypermethylation in preterm neonates included another key gene related to innate immunity, LBP, and hypermethylation of its promoter-reduced LBP mRNA expression within the first week in preterm vs. term newborns. Lipopolysaccharide-binding protein presents LPS to host cells through TLR4 (33), and we previously showed that the expression of LBP is positively correlated with bacterial adherence to the intestinal epithelium in preterm pigs (18). Reduced neonatal LBP expression following preterm birth may indicate for an immature gut immunity in preterm pigs, also reflected by reduced expression of genes in the TLR4 signaling pathway. In mice pups, the newborn intestine develops tolerance to LPS stimulation by expressing TLR4-dependent microRNA-146a, which represses the TLR4 signaling molecule IRAK1 and prevents nuclear factor κB (NF-κB) translocation (28). Correspondingly, we found that TLR4 and its accessory protein CD14 as well as microRNA-146a, but not MYD88, were down-regulated in the preterm intestine by day 5, probably leading to impaired LPS tolerance in an MYD88-independent manner. Consistently, the anti-inflammatory cytokine IL-10, which could be induced by microRNA-146a (34), was also less expressed in the preterm intestine by day 5. The proinflammatory cytokine IL-8 (a NF-κB target gene) was less expressed in the preterm intestine at birth but increased to a level comparable to that in term pigs already by day 5. In addition, C3, which is central for activation of the complement system, was persistently higher expressed in the preterm vs. term intestine during the first week. These results suggest that preterm birth is a risk factor for the immature intestine via an immature TLR4 signaling pathway, regardless of the initial feeding regimen (TPN or ENT) and at least partly regulated by epigenetic changes. It is important to note that both feeding regimens in the current study were NEC-protective (TPN or ENT with bovine colostrum). Only when fed infant formula, LBP expression increases more markedly in response to bacterial contact and results in much lower microRNA-146a, as well as much higher C3 levels, as shown previously (18). This helps to explain the well-known difference in NEC risk among TPN-, colostrum-, and formula-fed preterm newborn pigs and infants.

Unlike in the case of LBP, hypermethylation of NR3C1, encoding GR, was observed in the gene body with no difference in mRNA expression between newborn preterm and term pigs. Hypermethylation of NR3C1 in the preterm intestine may be associated with lack of cortisol stimulation before preterm birth, because methylation of this gene is sensitive to cortisol and stress responses (35). In both pigs and humans, circulating glucocorticoid levels increase markedly prior to term (36) to regulate final maturation of many organ functions, including the intestine (37, 38). A better understanding of the potential regulatory role of methylation in NR3C1 gene body is required.

While most newborn intestinal methylation defects in preterm pigs disappeared rapidly postnatally, novel preterm–term differences were identified, especially in relation to metabolic pathways in 5-day-old pigs fed TPN. These TPN-specific methylation changes covered 0.38% genome-wide cytosines, which were much more than that detected at birth (0.05%). Pathway enrichment analysis identified one gene, GCK (glucokinase), whose promotor hypomethylation likely upregulated the corresponding GCK transcription in TPN-treated preterm pigs. This may reflect a specific adaptation of the preterm TPN-fed intestine to facilitate glucose metabolism (relative to fat and amino acid metabolism). The inverse correlation with circulating GIP levels, an important sensor to luminal glucose (30), supports this hypothesis. In addition, protein–protein interaction analysis indicated that GCK might interact with PPARA (peroxisome proliferator-activated receptor α) and FABP1 (fatty acid binding protein 1), both of which are major regulators of fatty acid metabolism (39–41) and showed aberrant DNA methylation in TPN-fed preterm pigs. Thus, gene methylation of GCK may help to adapt intestinal nutrient metabolism in preterm neonates during the important transition from parenteral to enteral nutrition. Despite the marked feeding-dependent DNA methylation reprogramming in the first week of life, the intestinal DNA methylation in preterm neonates converges toward the pattern observed in term counterparts, with advancing postnatal age and transition to full ENT.

The present study used whole intestinal tissue samples, not isolated cell types (e.g., enterocytes) for DNA methylation analysis. This approach likely better represents the *in vivo* state of the intestine with many different cell types interacting during development and in response to feeding and diseases. On the other hand, this approach limited our ability to identify cell type–specific methylation changes associated with preterm birth and early feeding regimens. As DNA methylation is known to vary across cell types (42), changes in the intestinal cell composition may also contribute to DNA methylation changes in the intestinal tissue. However, as we did not observe any difference in the proportion of epithelial cells between preterm and term pigs along development, the reported DNA methylation differences are unlikely to be due to major changes in relative cell compositions.

In conclusion, the preterm intestine has a remarkable capacity to adapt to postnatal life, and this may involve highly dynamic gene methylation changes. At preterm birth, the intestinal epigenetic patterns may reflect incomplete fetal programming associated with immature morphology, digestive capacity and elevated risk of inflammation. The immature intestine has a high reprogramming capacity, involving both immunological and metabolic plasticity. The first week after preterm birth is an important window of opportunity, and early and gradual introduction of protective milk diets (e.g., mother's own milk or colostrum) may be important to facilitate optimal adaptation.

## Data Availability Statement

The data sets generated for this study can be found in the NCBI Gene Expression Omnibus (GEO) with accession GSE108284.

## Ethics Statement

The animal study was reviewed and approved by the Danish National Committee on Animal Experimentation.

## Author Contributions

XP and FG analyzed and interpreted the data. XP, FG, and PS were major contributors in writing the manuscript. TT took part in the main study design. All authors read and approved the final manuscript.

### Conflict of Interest

The authors declare that the research was conducted in the absence of any commercial or financial relationships that could be construed as a potential conflict of interest.

## References

[1] Beck S Wojdyla D Say L Betran AP Merialdi M Requejo JH. The worldwide incidence of preterm birth: a systematic review of maternal mortality and morbidity. Bull World Health Organ. (2010) 88:31–8. 10.2471/BLT.08.062554 20428351 PMC2802437

[2] Ward RM Beachy JC. Neonatal complications following preterm birth. BJOG. (2003) 110(Suppl 20):8–16. 10.1046/j.1471-0528.2003.00012.x 12763105

[3] Siggers RH Siggers J Thymann T Boye M Sangild PT. Nutritional modulation of the gut microbiota and immune system in preterm neonates susceptible to necrotizing enterocolitis. J Nutr Biochem. (2011) 22:511–21. 10.1016/j.jnutbio.2010.08.002 21193301

[4] Marlow N Wolke D Bracewell MA Samara M Group EPS. Neurologic and developmental disability at six years of age after extremely preterm birth. N Engl J Med. (2005) 352:9–19. 10.1056/NEJMoa041367 15635108

[5] Moster D Lie RT Markestad T. Long-term medical and social consequences of preterm birth. N Engl J Med. (2008) 359:262–73. 10.1056/NEJMoa0706475 18635431

[6] Wang G Divall S Radovick S Paige D Ning Y Chen Z. Preterm birth and random plasma insulin levels at birth and in early childhood. JAMA. (2014) 311:587–96. 10.1001/jama.2014.1 24519298 PMC4392841

[7] Kajantie E Strang-Karlsson S Hovi P Wehkalampi K Lahti J Kaseva N. Insulin sensitivity and secretory response in adults born preterm: the Helsinki Study of Very Low Birth Weight Adults. J Clin Endocrinol Metab. (2015) 100:244–50. 10.1210/jc.2014-3184 25303493

[8] Heird WC Gomez MR. Total parenteral nutrition in necrotizing enterocolitis. Clin Perinatol. (1994) 21:389–409. 10.1016/S0095-5108(18)30352-X 8070233

[9] Kudsk KA. Current aspects of mucosal immunology and its influence by nutrition. Am J Surg. (2002) 183:390–8. 10.1016/S0002-9610(02)00821-8 11975926

[10] Neu J. Gastrointestinal development and meeting the nutritional needs of premature infants. Am J Clin Nutr. (2007) 85:629S−34S. 10.1093/ajcn/85.2.629S 17284768

[11] Stoll B Horst DA Cui L Chang X Ellis KJ Hadsell DL. Chronic parenteral nutrition induces hepatic inflammation, steatosis, and insulin resistance in neonatal pigs. J Nutr. (2010) 140:2193–200. 10.3945/jn.110.125799 20980637 PMC2981005

[12] Sangild PT Thymann T Schmidt M Stoll B Burrin DG Buddington RK. Invited review: the preterm pig as a model in pediatric gastroenterology. J Anim Sci. (2013) 91:4713–29. 10.2527/jas.2013-6359 23942716 PMC3984402

[13] Sangild PT Siggers RH Schmidt M Elnif J Bjornvad CR Thymann T. Diet- and colonization-dependent intestinal dysfunction predisposes to necrotizing enterocolitis in preterm pigs. Gastroenterology. (2006) 130:1776–92. 10.1053/j.gastro.2006.02.026 16697741

[14] Bjornvad CR Thymann T Deutz NE Burrin DG Jensen SK Jensen BB. Enteral feeding induces diet-dependent mucosal dysfunction, bacterial proliferation, and necrotizing enterocolitis in preterm pigs on parenteral nutrition. Am J Physiol Gastrointest Liver Physiol. (2008) 295:G1092–103. 10.1152/ajpgi.00414.2007 18818317

[15] Shen RL Thymann T Ostergaard MV Stoy AC Krych L Nielsen DS. Early gradual feeding with bovine colostrum improves gut function and NEC resistance relative to infant formula in preterm pigs. Am J Physiol Gastrointest Liver Physiol. (2015) 309:G310–23. 10.1152/ajpgi.00163.2015 26138468

[16] Sangild PT. Gut responses to enteral nutrition in preterm infants and animals. Exp Biol Med. (2006) 231:1695–711. 10.1177/153537020623101106 17138756

[17] Willems R Krych L Rybicki V Jiang P Sangild PT Shen RL. Introducing enteral feeding induces intestinal subclinical inflammation and respective chromatin changes in preterm pigs. Epigenomics. (2015) 7:553–65. 10.2217/epi.15.13 26111029

[18] Pan X Gong D Gao F Sangild PT. Diet-dependent changes in the intestinal DNA methylome after introduction of enteral feeding in preterm pigs. Epigenomics. (2018) 10:395–408. 10.2217/epi-2017-0122 29587528

[19] Reik W. Stability and flexibility of epigenetic gene regulation in mammalian development. Nature. (2007) 447:425–32. 10.1038/nature05918 17522676

[20] Pan X Gong D Nguyen DN Zhang X Hu Q Lu H Early microbial colonization affects DNA methylation of genes related to intestinal immunity and metabolism in preterm pigs. DNA Res. (2018) 25:287:96 10.1093/dnares/dsy001 PMC6014285 29365082

[21] Andersen AD Sangild PT Munch SL van der Beek EM Renes IB Ginneken C. Delayed growth, motor function and learning in preterm pigs during early postnatal life. Am J Physiol Regul Integr Comp Physiol. (2016) 310:R481–92. 10.1152/ajpregu.00349.2015 26764054

[22] Hansen CF Thymann T Andersen AD Holst JJ Hartmann B Hilsted L. Rapid gut growth but persistent delay in digestive function in the postnatal period of preterm pigs. Am J Physiol Gastrointest Liver Physiol. (2016) 310:G550–60. 10.1152/ajpgi.00221.2015 26822913 PMC4836131

[23] Li Y Jensen ML Chatterton DE Jensen BB Thymann T Kvistgaard AS. Raw bovine milk improves gut responses to feeding relative to infant formula in preterm piglets. Am J Physiol Gastrointest Liver Physiol. (2014) 306:G81–90. 10.1152/ajpgi.00255.2013 24157971

[24] Meng M Li X Ge H Chen F Han M Zhang Y. Noninvasive prenatal testing for autosomal recessive conditions by maternal plasma sequencing in a case of congenital deafness. Genet Med. (2014) 16:972–6. 10.1038/gim.2014.51 24830326

[25] Li Y Nguyen DN de Waard M Christensen L Zhou P Jiang P. Pasteurization procedures for donor human milk affect body growth, intestinal structure, and resistance against bacterial infections in preterm pigs. J Nutr. (2017) 147:1121–30. 10.3945/jn.116.244822 28298536

[26] Guo H Zhu P Yan L Li R Hu B Lian Y. The DNA methylation landscape of human early embryos. Nature. (2014) 511:606–10. 10.1038/nature13544 25079557

[27] van der Flier LG Clevers H. Stem cells, self-renewal, and differentiation in the intestinal epithelium. Annu Rev Physiol. (2009) 71:241–60. 10.1146/annurev.physiol.010908.163145 18808327

[28] Chassin C Kocur M Pott J Duerr CU Gutle D Lotz M. miR-146a mediates protective innate immune tolerance in the neonate intestine. Cell Host Microbe. (2010) 8:358–68. 10.1016/j.chom.2010.09.005 20951969

[29] Nino DF Sodhi CP Hackam DJ. Necrotizing enterocolitis: new insights into pathogenesis and mechanisms. Nat Rev Gastroenterol Hepatol. (2016) 13:590–600. 10.1038/nrgastro.2016.119 27534694 PMC5124124

[30] Andersen DK Elahi D Brown JC Tobin JD Andres R. Oral glucose augmentation of insulin secretion. Interactions of gastric inhibitory polypeptide with ambient glucose and insulin levels. J Clin Invest. (1978) 62:152–61. 10.1172/JCI109100 659629 PMC371748

[31] Corpeleijn WE Kouwenhoven SM Paap MC van Vliet I Scheerder I Muizer Y. Intake of own mother's milk during the first days of life is associated with decreased morbidity and mortality in very low birth weight infants during the first 60 days of life. Neonatology. (2012) 102:276–81. 10.1159/000341335 22922675

[32] Yoo LI Chung DC Yuan J. LKB1–a master tumour suppressor of the small intestine and beyond. Nat Rev Cancer. (2002) 2:529–35. 10.1038/nrc843 12094239

[33] Lee CC Avalos AM Ploegh HL. Accessory molecules for Toll-like receptors and their function. Nat Rev Immunol. (2012) 12:168–79. 10.1038/nri3151 22301850 PMC3677579

[34] Luo X Han M Liu J Wang Y Luo X Zheng J. Epithelial cell-derived micro RNA-146a generates interleukin-10-producing monocytes to inhibit nasal allergy. Sci Rep. (2015) 5:15937. 10.1038/srep15937 26526003 PMC4630644

[35] Turner JD Alt SR Cao L Vernocchi S Trifonova S Battello N. Transcriptional control of the glucocorticoid receptor: CpG islands, epigenetics and more. Biochem Pharmacol. (2010) 80:1860–8. 10.1016/j.bcp.2010.06.037 20599772

[36] Fowden AL Valenzuela OA Vaughan OR Jellyman JK Forhead AJ. Glucocorticoid programming of intrauterine development. Domest Anim Endocrinol. (2016) 56(Suppl):S121–32. 10.1016/j.domaniend.2016.02.014 27345310

[37] Arsenault P Menard D. Influence of hydrocortisone on human fetal small intestine in organ culture. J Pediatr Gastroenterol Nutr. (1985) 4:893–901. 10.1097/00005176-198512000-00008 4067777

[38] Sangild PT Sjostrom H Noren O Fowden AL Silver M. The prenatal development and glucocorticoid control of brush-border hydrolases in the pig small intestine. Pediatr Res. (1995) 37:207–12. 10.1203/00006450-199502000-00014 7731759

[39] Bunger M van den Bosch HM van der Meijde J Kersten S Hooiveld GJ Muller M. Genome-wide analysis of PPARalpha activation in murine small intestine. Physiol Genomics. (2007) 30:192–204. 10.1152/physiolgenomics.00198.2006 17426115

[40] Piomelli D. A fatty gut feeling. Trends Endocrinol Metab. (2013) 24:332–41. 10.1016/j.tem.2013.03.001 23567058 PMC3778443

[41] Gajda AM Storch J. Enterocyte fatty acid-binding proteins (FABPs): different functions of liver and intestinal FABPs in the intestine. Prostaglandins Leukot Essent Fatty Acids. (2015) 93:9–16. 10.1016/j.plefa.2014.10.001 25458898 PMC4323920

[42] Roadmap Epigenomics C Kundaje A Meuleman W Ernst J Bilenky M Yen A Integrative analysis of 111 reference human epigenomes. Nature. (2015) 518:317–30. 10.1038/nature14248 25693563 PMC4530010

